# Stress Calculations of Heat Storage Tanks

**DOI:** 10.3390/ma15051647

**Published:** 2022-02-22

**Authors:** Weronika Wiśniewska, Robert Matysko

**Affiliations:** Laboratory of the Heat Processes Control Systems, Centre of Heat and Power Engineering, Turbine Department, The Szewalski Institute of Fluid-Flow, Machinery Polish Academy of Sciences, Fiszera 14 St., 80-231 Gdansk, Poland; matyskor@imp.gda.pl

**Keywords:** stress calculation of tanks, pressure tank, FEA, FEM, designing tanks

## Abstract

Stress calculations are necessary to determine the feasibility and profitability of a heat storage tank’s construction. The article presented normative methods of stress calculations for a heat storage tank. Results were verified by finite element analysis. These stress calculations enabled us to determine wall and weld thickness. The calculations were made on the example of a tank with a nominal pressure of 10 bar. The work undertook an extensive analysis of the stresses occurring in a pressure tank, described the finite element method and showed examples of ways in which it could be used. During stress analysis, three types of materials were compared: carbon steel St0 (S185), stainless steel (304) and boiler steel (P 265 GH). A brief overview of types of thermal energy storages was also provided.

## 1. Introduction

The world is currently facing a climate crisis. Construction is an industrial field that contributes significantly to the consumption of fossil fuels. About 40% of energy is used in mid-latitude and high-latitude countries to provide hot water. According to forecasts, this value could increase to 50% by 2050, leading to increasing air pollution [1]. Obtaining energy from renewable sources, for example, from solar collectors, is a promising solution to the above problem. However, the generation of energy from renewable sources has some limitations. One major disadvantage is that energy is not produced continuously. For this reason, thermal energy storage systems are seeing increased use in the heating systems of single-family houses. The most commonly used thermal energy storage solutions are water heat storage tanks, which serve as parts of heat distribution systems in domestic hot water and central heating systems.

A water heat storage tank, during operation, is exposed to various loads as a result of complex stresses. Unsealing the tank could lead not only to the loss of system functionality but also to its destruction—perhaps even risking the health of people nearby. Thus, carrying out stress calculations and verifying their results is an important step in designing a tank.

During the tank designing process, stress calculations are often performed using normative methods or finite element analysis. This article compared the results of both these methods.

## 2. The Concept of Thermal Energy Storage

**Figure 1:**
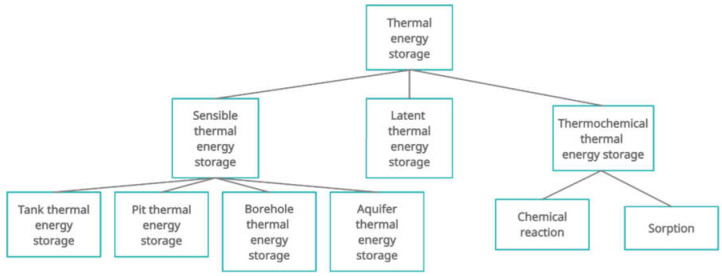
Types of thermal energy storages [1,2].

Thermal energy storage solutions, as described above, perform as regulators of heat distribution in domestic hot water and central heating systems during periodic differences between supply and demand. Regardless of the type, thermal energy storages should have a high heat capacity. They should also be environmentally friendly and should not be very corrosive. The system should be both mechanically and chemically stable. The cost of the investment and the area it occupies are also important. Three types of thermal energy storages can be distinguished: sensible thermal energy storage, latent thermal energy storage and thermochemical thermal energy storage (Figure 1) [1,2].

Latent heat storage consists of storing the thermal energy from the phase change at a constant temperature. It is a purely physical process, with no chemical reactions. Recently, methods for modification of sensible heat storages through the addition of phase change materials have been increasingly researched [3]. Thermochemical thermal energy storages, as the name suggests, generate thermal energy from reversible chemical reactions [2].

Sensible thermal energy storage is based on raising the temperature of the storage medium (most often water, but sometimes oil, sand or rock deposits). Storing thermal energy is proportional to the temperature difference, the mass flow of the medium and the specific heat. Water used for heating and cooling buildings has a high heat capacity and is easily available [4]. However, new studies on improving heat storage systems by adding phase change materials are still being carried out.

The technology behind heat storage tanks is among the oldest and most-used in both small buildings and large-scale constructions. These tanks can be used for short-term or long-term thermal energy storage. They have several advantages, including relatively high stratification, high heat capacity, ease of installation and a lack of special requirements regarding geological conditions. However, systems with heat storage tanks lose thermal energy to the environment and carry the risk of corrosion or potential leakage [1].

Despite many years of research on heat storage tanks, new works are still regularly carried out, primarily focused on optimizing the structure of the tank and the entire system. The work in [5] described the three-finned-coil hot water storage tank, which recovered heat from the air-conditioning system and had a special device to maintain higher thermal stratification. W. Gaggioli et al. [6] published an article about an innovative thermal energy storage system based on a single tank containing molten salts and an integrated steam generator.

However, most research has focused on the geometry of the tank. The shape of the tank should enable the highest possible thermal stratification [7], associated with limiting the mixing of hot and cold water. The design of the tank should also minimize cold water weight and heat loss. For this purpose, tanks with various geometric parameters have been tested, including wall thickness, different insulation, shape factor and inlets and outlets (e.g., location, shape or type of diffuser) [3].

Exemplary works about optimizing the tank geometry include Tao Li et al. in [8], and Abdulrahman Dahash et al. in [9], who wrote about the role of selecting the optimal tank volume. Additionally, in [9], the ratio of height to diameter (h/d) was specified. The authors of [10] dealt with the subject of different tank shapes in order to find the optimal one. The authors described the influence of various inlet shapes on the efficiency of the tank in [11].

The currently available literature, as mentioned above, covers topics related to the optimization of the heat storage system and the geometry of the tank. However, in order to analyze whether a solution is feasible and profitable, stress calculations of the tank must be carried out, allowing for the selection of the optimal tank wall thickness while considering the operating pressure. In new solutions with solar plants, because of the possibility of obtaining temperatures above 120 °C, components—including hot water tanks—must have high mechanical strength. The subject of this work was stress calculations of heat storage tanks.

## 3. Stress Calculations of the Tank

While designing a heat storage tank, stress calculations must be carried out to select the optimal thickness of the wall and welds. Stress calculations of pressure vessels consist of comparing the stresses in the tank to the stress limits of the used material:

(1) $${\left(\sigma_{1}-\frac{\sigma_{2}}{z}\right)}^{2}+{\left(\sigma_{1}-\sigma_{3}\right)}^{2}+{\left(\frac{\sigma_{2}}{z}-\sigma_{3}\right)}^{2}\leq 2k^{2},$$

where:

- $\sigma_{1}$—hoop stresses [Pa],
- $\sigma_{2}$—longitudinal stresses [Pa],
- $\sigma_{3}$—radial stresses [Pa],
- k—allowable stresses in selected material [Pa],

(2) $$\sigma_{1}=\frac{p_{0}D}{2g},$$

(3) $$\sigma_{2}=\frac{p_{0}D^{2}}{4Dg},$$

(4) $$\sigma_{3}=\frac{p_{0}}{2},$$

where:

- g—wall thickness [m],
- $p_{0}$—operating pressure in tank [Pa],
- D—internal diameter of tank [$m^{2}$].

**Figure 2:**
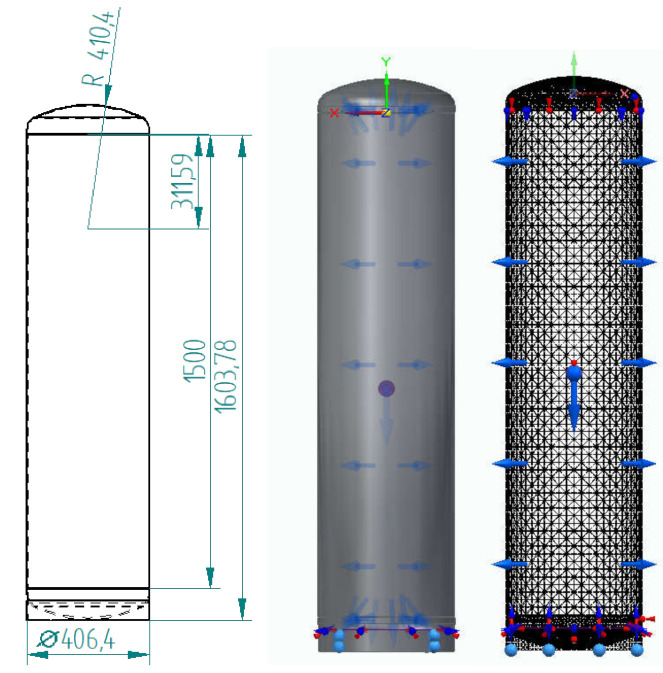
Geometry and mesh for stress analysis of a heat storage tank.

The calculations shown below used the example of a tank with a maximum operating pressure of 10 bar and a temporary pressure that could not exceed 16 bar. The geometric parameters are shown in Figure 2.

Various materials for tanks are used, from plastic to steel alloys. Depending on the material, heat storage tanks may be equipped with magnesium anode systems to increase the corrosion resistance of components made of susceptible carbon steel.

The calculations were made using classic and standardized engineering methods (for tanks made of 304 stainless steel and carbon St0 and the boiler P 265 GH). Then, the results were verified with the use of FEM algorithms (for the tank made of 304 stainless steel).

### 3.1. The Thickness of the Tank Wall Due to the Classic Normative Requirements

The Polish Office of Technical Inspection [12] provided the relationship to calculate the wall thickness of the tank:

(5) $$g\leq \frac{p_{0}D}{\frac{2.3}{\alpha }kz-p_{0}},$$

where:

- g—wall thickness [m],
- $p_{0}$—operating pressure in tank [Pa], estimated at maximum temporary pressure $p_{0}=1.6\;\mathrm{MPa}$,
- D—internal diameter of tank [m],
- k—allowable stresses in selected material [Pa],
- z—design stress coefficient in the longitudinal direction,
- α—coefficient depending on β.

The coefficient β should be estimated based on expected wall thickness of the tank:

(6) $$\beta =\frac{D_{o}}{D_{i}},$$

where:

- $D_{o}$—outer diameter of the tank [$m^{2}$],
- $D_{i}$—internal diameter of the tank [$m^{2}$].

Then, having already calculated the β coefficient, we can read the value of the coefficient α from Table 1.

In order to obtain the lowest possible value of the α coefficient, the condition (7) was checked for value of β equal to 1.4:

(7) $$\beta =\frac{D_{i}+2g}{D_{i}}.$$

The maximum wall thickness for α=1 and g=80 mm. Considering the geometry of the tank, the wall will be a few mm thick, so this condition will certainly be met. Thus, the coefficient α=1. The value of the wall thickness g=80 mm is nonbinding to the calculations below.

The design stress coefficient in the longitudinal direction z depends on whether the element has been welded. If not, the coefficient z is equal to one. Otherwise, it depends on the category of the welded joint and the coefficient with which the factory has been allowed to make joints in pressure vessels. The factory will not manufacture the designed tank. Due to the lack of data, z was estimated to 0.7.

Allowable stress k depends on the material of the tank, specifically on its yield point and the operating temperature (data for the materials are given in Table 2):

(8) $$k=\frac{Re_{t_{0}}}{x},$$

(9) $$Re_{t_{0}}=A*Re,9$$

(10) $$A=1.019-0.09\left(\frac{t_{0}}{100}\right)-0.018{\left(\frac{t_{0}}{100}\right)}^{2},$$

where:

- $Re_{t_{0}}$—lowest guaranteed yield point at the operating temperature of the component [Pa],
- x—safety coefficient, depending on the material,
- Re—yield point [Pa],
- A—coefficient of stress reduction at the operate temperature,
- $t_{0}$—maximum operate temperature [°C], estimated to $t_{0}=90\;{}^{\circ}C$.

The wall thicknesses for selected materials and the tank parameters are given below in Table 3.

These results are often calculated with an excess because, while estimating the values of safety coefficients, correction coefficients, etc., committees prefer to overestimate values. In this way, the tank is resistant to all threats, even the least likely ones.

### 3.2. The Thickness of Weld Due to the Classic Normative Requirements

During welding, complex thermal phenomena occur that result in structural changes. While the material is heated to the melting point by a gas flame, electric arc, plasma beam, electron beam or laser beam, it expands and contracts. Thermomechanical properties of metals influence the kinetics of stresses and thermal deformations and, consequently, deformations of the structure. The deformation and stress also depend on the selected welding technology [16].

The material strength of the weld depends mainly on the quality of its execution, i.e., on the skills and training of the welder, or the use of a properly programmed automat [17]. The butt weld thickness is estimated as the thickness of the thinnest element. On the other hand, the fillet weld should be at least 3 mm thick. If it is possible, it should be in the range:

(11) 0.2g≤a≤0.7g,

where:

- g—wall thickness [m],
- a—fillet weld thickness [m].

The thicknesses of the welds for selected materials are presented below in Table 4.

Various publications and websites present different dependencies for stress calculations. It is worth carrying out finite element analysis to verify the results. This can be achieved using commercial software for designing, i.e., computer-aided design (CAD) software.

## 4. Finite Element Method

At the beginning of the 20th century, Lord Rayleigh, Ritz and Galerkin used trial function to approximate solutions for differential equations. This was the basis for the creation of the finite element method. In the 1940s, Courant introduced the concept of piecewise-continuous functions in a subdomain. Additionally, in the 1940s, the displacement method, which corresponded to the finite element method, was developed. The phrase finite element was first used by Clough in the 1960s and is still valid today [18]. The finite element method has been continuously developing since then, especially in the context of finite element analysis.

The finite element method consists of dividing a continuous geometric model into finite elements, combining into so-called nodes. As a result, a discrete geometric model with a finite number of degrees of freedom can be obtained. During FEM calculations, other physical quantities appearing in the system are also discretized [19].

A finite element is a simple geometric figure (flat or spatial) for which points called nodes have been defined, and some interpolation functions have been used to describe the distribution of the analyzed quantity inside and on its sides. After dividing the object into finite elements and determining interpolation functions, differential equations describing the phenomena under study are transformed into algebraic equations using so-called weight functions. Based on the equations of the finite element method, the system of equations is assembled, i.e., the values of the coefficients of the unknowns and the corresponding values of the right-hand sides are calculated. If the problem to be solved is inconsistent, the initial conditions are additionally used in calculating the right-sided values. The number of equations in the system is equal to the number of nodes multiplied by the number of node degrees of freedom, i.e., the number of unknowns in a single node. Boundary conditions are introduced to the system of equations by making appropriate modifications to the matrix of coefficients of the system of equations and the vector of right-hand sides. Then, the system of equations can be solved, as a result of which the values of the physical quantities in the nodes can be obtained.

### 4.1. Finite Element Analysis

FEA owes its popularity to the ease with which the user can change the initial conditions and the accuracy of the calculations. FEA has found application in many fields of science where it is necessary to approximate the basic differential equations of mathematical physics. This method is used in deformable body mechanics, fluid mechanics, acoustics, electromagnetism, atomic physics and even in medicine.

FEA comprises three steps: preprocessing, finite element solution and proprocessing. During preprocessing, the tested object should be designed (its geometry and material selected), and the existing loads and boundary conditions should be defined. Finite element solution starts with selecting solution parameters, such as type of finite element and mesh. Then, after receiving the solution, it is necessary to decide whether the accuracy of the solution is sufficient. Post-processing is based on the analysis of the obtained results and their subsequent presentation as graphics, charts and tables [20].

### 4.2. Stress Analysis

From the beginning, one of the main applications of FEA has been stress analysis. Many works in the available literature are related to this.

Finite element analyses can calculate failure time, e.g., related to corrosion or shape. In the work in [21], FEA was used to predict defects subjected to internal pressure and axial compressive stress of corroded pipelines, and was verified by theoretical calculations. Article [22] was about nonlinear FEA of the sustained stress indices of a 90 degree pipe elbow. Paper [23] described the model of corrosion damage process, developed with cellular automata (CA) and finite element analysis (FEA), carried out to account for the mechanical components resulting from the stress concentration effects of the corrosion defect (pit).

Finite element analysis also helps in assessing the broadly defined safety of industrial processes. Eren Kayaoglu, Ozlem Salman and Adem Candas [24] researched stress and deformation of an elevator safety gear brake block. The work [25] used FEA to analyze steel coil holder fixtures to maintain production continuity.

In FEM models, it is possible to take into account thermal parameters and their in-fluence on the wall deformation. Other models of the influence of temperature on stress-strain relations have also been explored. The dependency on strain rate and temperature has been described well, for example, in the Anand model by Xu Long, Zubin Chen, Wenjie Wang, Yonghui Fu and Yanpei Wu [26].

Finite element analysis can also be used for weld analysis. This method was used in [16], the purpose of which was to develop an inexpensive, fast and effective method of welding stub pipes to the shell of a cylindrical tank without the formation of large shell distortions. A description of how to prepare the FEA model for this application can be found in [27].

Finite element analysis of stress would not exist without an applicable model of phenomena taking place in the researched object. The authors of [18,20,28,29] worked to describe the phenomena using equations. Deformations and stresses can be defined by formulas (12) and (13).

(12) $$\left\{\varepsilon \right\}=\left\{\begin{array}{c} \varepsilon_{x}\\ \varepsilon_{y}\\ \begin{array}{c} \varepsilon_{z}\\ \gamma_{xy}\\ \begin{array}{c} \gamma_{xz}\\ \gamma_{yz} \end{array} \end{array} \end{array}\right\}=\left\{\begin{array}{c} \frac{\partial u}{\partial x}\\ \frac{\partial v}{\partial y}\\ \begin{array}{c} \frac{\partial w}{\partial z}\\ \frac{\partial u}{\partial y}+\frac{\partial v}{\partial x}\\ \begin{array}{c} \frac{\partial u}{\partial z}+\frac{\partial w}{\partial x}\\ \frac{\partial v}{\partial z}+\frac{\partial w}{\partial y} \end{array} \end{array} \end{array}\right\}=\left[\begin{array}{c} \begin{array}{ccc} \frac{\partial }{\partial x} & 0 & 0\\ 0 & \frac{\partial }{\partial y} & 0\\ 0 & 0 & \frac{\partial }{\partial z} \end{array}\\ \begin{array}{ccc} \frac{\partial }{\partial y} & \frac{\partial }{\partial x} & 0\\ \frac{\partial }{\partial z} & 0 & \frac{\partial }{\partial x}\\ 0 & \frac{\partial }{\partial z} & \frac{\partial }{\partial y} \end{array} \end{array}\right]\left\{\begin{array}{c} u\\ v\\ w \end{array}\right\}=\left[L\right]\left\{\begin{array}{c} u\\ v\\ w \end{array}\right\},$$

(13) $$\left\{\sigma \right\}=\left\{\begin{array}{c} \sigma_{x}\\ \sigma_{y}\\ \begin{array}{c} \sigma_{z}\\ \tau_{xy}\\ \begin{array}{c} \tau_{xz}\\ \tau_{yz} \end{array} \end{array} \end{array}\right\}=\frac{E}{\left(1+v\right)\left(1-2v\right)}\left[\begin{array}{cc} \begin{array}{ccc} 1-v & v & v\\ v & 1-v & v\\ v & v & 1-v \end{array} & \begin{array}{ccc} 0\;\;\; & \;\;\;0\;\;\;\;\; & 0\\ 0\;\;\; & 0\;\; & 0\\ 0\;\;\; & \;0\;\;\; & 0 \end{array}\\ \begin{array}{ccc} \;\;\;0\;\;\;\; & \;\;\;0\;\;\; & \;\;\;\;\;0\;\;\;\\ 0\; & 0 & \;\;0\\ 0\; & 0 & \;\;0 \end{array} & \begin{array}{ccc} \frac{1-2v}{2} & 0 & 0\\ 0 & \frac{1-2v}{2} & 0\\ 0 & 0 & \frac{1-2v}{2} \end{array} \end{array}\right]\left\{\varepsilon \right\}=\left[D\right]\left\{\varepsilon \right\},$$

where:

- E—elastic modulus,
- v—Poisson’s ratio.

Assuming that element have M nodes, the displacement elements after discretizing are described by Equations (14)–(16).

(14) $$u\left(x,\;y,\;z\right)=\sum_{i=1}^{M}N_{i}\left(x,\;y,\;z\right)u_{i},$$

(15) $$v\left(x,\;y,\;z\right)=\sum_{i=1}^{M}N_{i}\left(x,\;y,\;z\right)v_{i},$$

(16) $$w\left(x,\;y,\;z\right)=\sum_{i=1}^{M}N_{i}\left(x,\;y,\;z\right)w_{i},$$

where:

- $N_{i}$—interpolation function.

It also can be presented as a matrix:

(17) $$\left\{\begin{array}{c} u\\ v\\ w \end{array}\right\}=\left[\begin{array}{ccc} \left[N\right] & \left[0\right] & \left[0\right]\\ \left[0\right] & \left[N\right] & \left[0\right]\\ \left[0\right] & \left[0\right] & \left[N\right] \end{array}\right]\left\{\delta \right\},$$

where:

- $N=\left[\begin{array}{ccc} N_{1} & N_{2} & \begin{array}{cc} \ldots & N_{M} \end{array} \end{array}\;\right]$,
- $\left\{\delta \right\}={\left[\begin{array}{ccc} u_{1} & u_{2} & \begin{array}{ccc} \ldots & u_{M} & \begin{array}{ccc} v_{1} & v_{2} & \begin{array}{ccc} \ldots & v_{M} & \begin{array}{ccc} w_{1} & w_{2} & \begin{array}{cc} \ldots & w_{M} \end{array} \end{array} \end{array} \end{array} \end{array} \end{array}\right]}^{T}$.

Total potential energy of element may be defined as:

(18) $$\prod =U_{e}-W=\frac{1}{2}{\int \int \int }_{V}{\left\{\varepsilon \right\}}^{T}\left[D\right]\left\{\varepsilon \right\}dV-{\left\{\delta \right\}}^{T}\left\{f\right\},$$

where:

- $\left\{f\right\}={\left[\begin{array}{ccc} f_{1x} & f_{2x} & \begin{array}{ccc} \ldots & f_{Mx} & \begin{array}{ccc} f_{1y} & f_{2y} & \begin{array}{ccc} \ldots & f_{My} & \begin{array}{ccc} f_{1z} & f_{2z} & \begin{array}{cc} \ldots & f_{Mz} \end{array} \end{array} \end{array} \end{array} \end{array} \end{array}\right]}^{T}$—the element nodal force vector.

The maximum shear stress theory and the distortion energy theory are methods used to determine the critical load. In a three-dimensional model, the principal stresses σ_1_, σ_2_ and σ_3_ are calculated as the roots of the cubic equation represented by the determinant (19).

(19) $$\left|\begin{array}{ccc} \sigma_{x}-\sigma & \tau_{xy} & \tau_{xz}\\ \tau_{xy} & \sigma_{y}-\sigma & \tau_{yz}\\ \tau_{xz} & \tau_{yz} & \sigma_{z}-\sigma \end{array}\right|=0$$

The maximum shear stress components are defined by formula (20):

(20) $$\tau_{max}=\max\left(\frac{\left|\sigma_{1}-\sigma_{2}\right|}{2},\;\frac{\left|\sigma_{1}-\sigma_{3}\right|}{2},\;\frac{\left|\sigma_{2}-\sigma_{3}\right|}{2}\right)=\frac{S_{y}}{2}=S_{ys},$$

where:

- $S_{y}$—tensile yield strength [Pa],
- $S_{ys}$—yield strength in shear [Pa].

To determine critical load by the distortion energy theory, a difference between total elastic strain energy and strain energy resulting from hydrostatic stresses must be calculated, as given below:

(21) $$U_{e}=\frac{1}{2}{\int \int \int }_{V}\left(\sigma_{1}\varepsilon_{1}+\sigma_{2}\varepsilon_{2}+\sigma_{3}\varepsilon_{3}\right)dV=\frac{1}{2E}\left[\sigma_{1}{}^{2}+\sigma_{2}{}^{2}+\sigma_{3}{}^{2}-2v\left(\sigma_{1}\sigma_{2}+\sigma_{1}\sigma_{3}+\sigma_{2}\sigma_{3}\right)\right]V,$$

(22) $$U_{hyd}=\frac{3{\frac{\left(\sigma_{1}+\sigma_{2}+\sigma_{3}\right)}{3}}^{2}}{2E}\left(1-2v\right)V=\frac{3\sigma_{avg}{}^{2}}{2E}\left(1-2v\right)V,$$

(23) Ud=Ue−Uhyd=1+v3Eσ1−σ22+σ1−σ32+σ2−σ32212V=1+v3ESy2V,

where:

- $U_{d}$—distortion energy [J],
- $U_{e}$—total elastic strain energy [J],
- $U_{hyd}$—strain energy resulting from hydrostatic stresses [J].

Based on the above equations, failure yielding is determined.

(24) σ1−σ22+σ1−σ32+σ2−σ32212≥Sy,

The formulas given above are used for stress analysis by finite element analysis in dedicated software.

## 5. Verification of the Calculation of Traditional Stress Methods Using the FEM Method

The selection of tank wall thickness is an issue that involves legal liability in case of leakage or disruption. In carrying out stress calculations of a tank with the assumption that the material of the tank walls will be exposed only to simple strength (tensile), as is in the classical engineering approach [30,31], it is impossible to verify the real combined stresses to which the walls of the tank may be exposed. Complex stresses were described by the Huber–Mises hypothesis, according to the formulae:

(25) $$\sigma_{red}=\sqrt{\frac{{\left(\sigma_{x}-\sigma_{y}\right)}^{2}+{\left(\sigma_{y}-\sigma_{z}\right)}^{2}+{\left(\sigma_{z}-\sigma_{x}\right)}^{2}}{2}+3\left(\tau_{xy}^{2}+\tau_{yz}^{2}+\tau_{zx}^{2}\right)},$$

(26) $$\sigma_{red}<k,$$

(27) $$k=\frac{\sigma_{d}}{x},$$

where:

- $\sigma_{red}$—complex stresses [Pa],
- $\sigma_{x}$—normal stresses [Pa],
- $\tau_{xy}$—tangential (shear) stresses [Pa],
- $\sigma_{d}$—destructive stresses [Pa],
- k—permissible stresses of the material depending on estimated safety coefficient x in relation to dangerous stresses (yield point [18], maximum destructive stress [30]).

**Figure 3:**
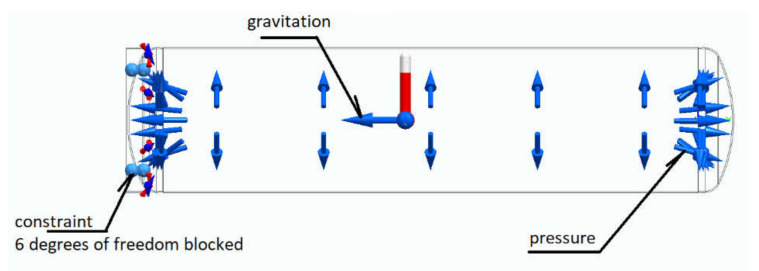
DOF and boundary conditions.

The classic method, because of its applied assumptions and simplifications and the discretionary estimated safety coefficients, may increase the amount of risk, especially when the tank is designed in such a way as to optimize material costs. If the tank wall thickness as calculated by the classical engineering method is too thin, the tank wall may fail if the yield point and maximum tensile stress limits are exceeded. Additional effects related to complex stresses that occur in tanks designed with the classical method are usually considered by a safety coefficient. The classical method of verifying the appropriate wall thickness of the tank involves doing experimental tests that could destroy the tank. However, due to the high cost of strain gauge test equipment (which should be prepared for various tank diameters from scratch), it is a relatively expensive method. Another method of verifying whether the tank wall thickness is correctly selected and does not exceed the stress limits is finite element analysis. DOF and boundary conditions are shown below in Figure 3.

During FEA, the computational geometry and the computational mesh size must be adopted. To perform calculations, the Galerkin integration method was used over nodes generated by the Delaunay triangulation method.

For the stress calculations of the tank, the numerical code NX Nastran (2020.1) was used. The material was 304 stainless steel. According to the classical calculations, the optimal wall thickness was 3 mm and the operating pressure equaled 10 bar. Table 5 presents the parameters of the steel used for the calculations.

### 5.1. The Galerkin Method

**Figure 4:**
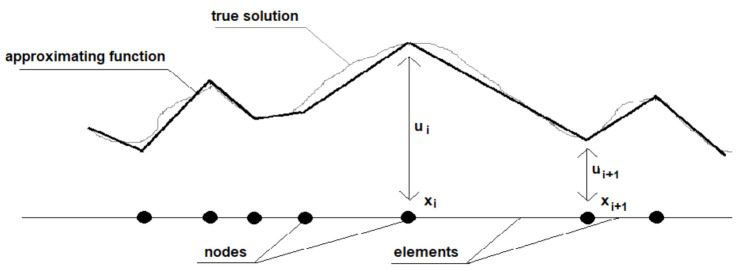
Function approximation.

The Galerkin method converts a continuous operator problem, such as a differential equation, to a discrete formula by applying linear constraints determined by finite sets of basic functions. In the finite element method, the Galerkin method is used to approximation function, e.g., shape function [28]. The method is presented schematically in Figure 4.

Approximation function using the Galerkin method begins with multiplying the differential equation by a weight function. Next, the internal should be formulated over the whole domain or, if necessary, by parts, to reduce the order. After that, the order of interpolation and corresponding shape function $H_{i}$ with trial function $u=\tilde{u}\left(x\right)=\overset{m}{\underset{i}{\sum }}H_{i}u_{i}$ must be chosen. Finally, as created by evaluation integrals, the system of equations in the unknown $u_{i}$’s must be solved. The procedure is described in the equations below.

(28) $$u=c_{1}x+c_{2}$$

(29) $$u\left(x_{i}\right)=c_{1}x_{i}+c_{2}=u_{i}$$

(30) $$u\left(x_{i+1}\right)=c_{1}x_{i+1}+c_{2}=u_{i+1}$$

(31) $$c_{1}=\frac{u_{i+1}-u_{i}}{x_{i+1}-x_{i}}$$

(32) $$c_{2}=\frac{u_{i}x_{i+1}-u_{i+1}x_{i}}{x_{i+1}-x_{i}}$$

(33) $$u=H_{1}\left(x\right)u_{i}+H_{2}\left(x\right)u_{i+1}$$

(34) $$H_{1}\left(x\right)=\frac{x_{i+1}-x}{x_{i+1}-x_{i}}$$

(35) $$H_{2}\left(x\right)=\frac{x-x_{i}}{x_{i+1}-x_{i}}$$

The equation may approximate function over each interval.

(36) $$\tilde{u}\left(x\right)=\begin{array}{c} H_{1}\left(x\right)\tilde{u_{1}}+H_{2}\left(x\right)\tilde{u_{2}}\\ H_{1}\left(x\right)\tilde{u_{2}}+H_{2}\left(x\right)\tilde{u_{3}}\\ \vdots \\ H_{1}\left(x\right)\tilde{u_{N}}+H_{2}\left(x\right)\tilde{u_{N+1}} \end{array}$$

The shape functions takes certain values on the *_i_* node.

(37) $$H_{1}\left(x_{i}\right)=1,\;\;H_{1}\left(x_{i+1}\right)=0,\;\;H_{2}\left(x_{i}\right)=0,\;\;H_{2}\left(x_{i+1}\right)=1$$

Therefore, the sum of the shape functions is equal to unity.

(38) $$\overset{2}{\underset{i=1}{\sum }}H_{i}\left(x\right)=1$$

### 5.2. The Delaunay Triangulation Method

**Figure 5:**
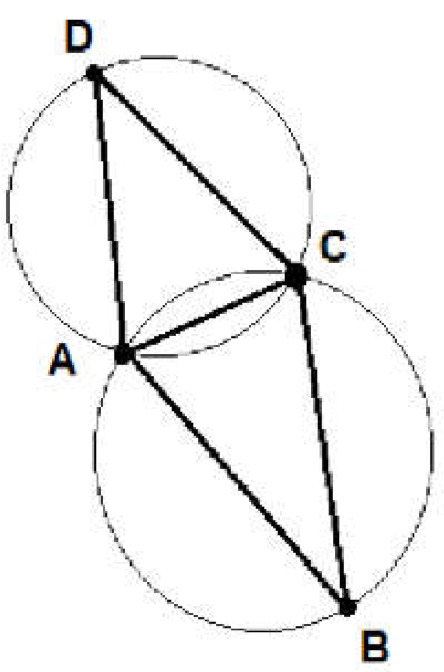
The Delaunay triangulation method.

Triangulation may be defined as connecting points using straight lines as many times as possible without crossing two segments. Delaunay triangulation is possible if the points in a set have generic positions (i.e., no three are collinear and no four lie in the same circle). The Delaunay triangulation is created by choosing the diagonal, which gives the largest minimum angle for six interior angles in the two triangles. Moreover, the circumcircle of any triangle contains no other vertices [32]. In addition, the union of all simplices produces the convex hull [33]. Another feature of the Delaunay triangulation is that it has the largest minimum angles among all triangulations. The Delaunay triangulation may be implemented using many algorithms as incremental algorithms, sweepline algorithms, local improvement algorithms or divide-and-conquer algorithms. The Delaunay triangulation method is one of most common methods of mesh generation and it is still being developed as of the time of this writing. The method is presented schematically in Figure 5.

### 5.3. Results

**Figure 6:**
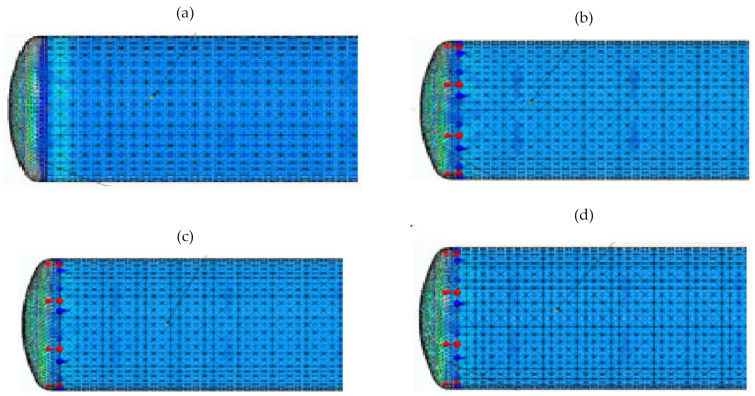
Complex stresses when a wall thickness is equal to 4 mm: (**a**) when pressure p=20 bar stresses for the shell $\sigma_{red}=86.6\;\mathrm{MPa}$, for the bottom $\sigma_{red}=185\;\mathrm{MPa}$; (**b**) when pressure p=15 bar, stresses for the shell $\sigma_{red}=65\;\mathrm{MPa}$, for the bottom $\sigma_{red}=146\;\mathrm{MPa}$; (**c**) when pressure  p=10 bar, stresses for the shell $\sigma_{red}=42.6\;\mathrm{MPa}$, for the bottom $\sigma_{red}=96.6\;\mathrm{MPa}$; (**d**) when pressure p=5 bar, stresses for the shell $\sigma_{red}=21.3\;\mathrm{MPa}$, for the bottom $\sigma_{red}=42.2\;\mathrm{MPa}.$

**Figure 7:**
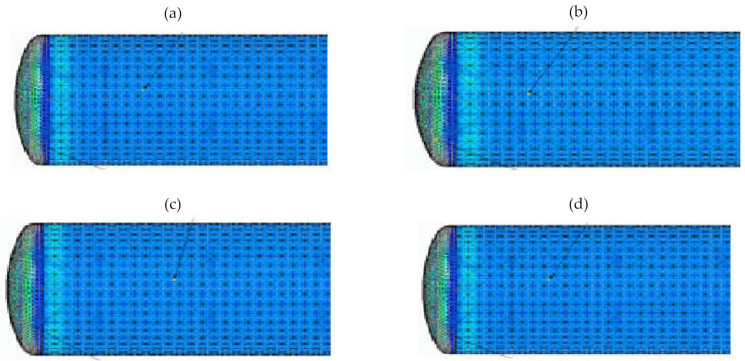
Complex stresses when a wall thickness is equal to 3 mm: (**a**) when pressure p=20 bar, stresses for the shell $\sigma_{red}=112\;\mathrm{MPa}$, for the bottom $\sigma_{red}=192\;\mathrm{MPa}$; (**b**) when pressure p=15 bar, stresses for the shell $\sigma_{red}=84.3\;\mathrm{MPa}$, for the bottom $\sigma_{red}=132\;\mathrm{MPa}$; (**c**) when pressure p=10 bar, stresses for the shell $\sigma_{red}=58.1\;\mathrm{MPa}$, for the bottom $\sigma_{red}=87\;\mathrm{MPa}$; (**d**) when pressure p=5 bar, stresses for the shell $\sigma_{red}=29\;\mathrm{MPa}$, for the bottom $\sigma_{red}=46\;\mathrm{MPa}.$

Figure 6 shows the results of calculations of the complex stresses for various pressure values in the tank in the range from 5 to 20 bar, when a tank wall thickness is equal to 4 mm. Figure 7 presents the results when a tank wall thickness is equal to 3 mm.

When analyzing the results of calculations for complex stresses, it was noted that the tank—which was designed using classical methods, with nominal pressure of 10 bar and a selected wall thickness of 3 mm—transferred even higher pressures without exceeding the yield point of 255 MPa. The safety coefficient for the shell was estimated at a pressure of p=20 bar. Thus, x=2.27:

$$x=\frac{k}{\sigma_{red}}.$$

The calculations showed a significant increase in the complex stresses on the tank bottom. Therefore, the effects of this stress on the material strength of the tank were also analyzed. In this case, when wall thickness was equal to 3 mm, the stress value at a nominal pressure of 10 bar was safe and amounted to 87 MPa. On the other hand, operation at higher pressure, e.g, 20 bar, significantly increased the stress, nearly reaching the yield point but not exceeding it. The tank was able to temporarily transfer stresses higher than 10 bar without the risk of damaging its bottom.

**Figure 8:**
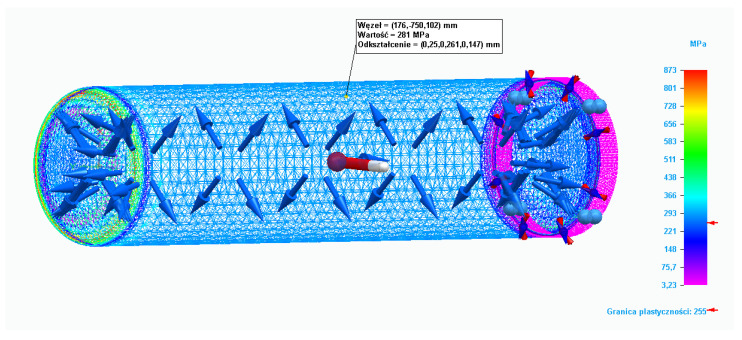
Complex stresses when pressure load is equal to 50 bar.

Stress calculations were carried out in the Solid Edge (Premium 2021 by Siemens purchased from Cador Consulting Sp. z o. o., Gdynia, Poland) commercial software, in which the ways of editing the calculation mesh types were limited. During FEM analysis, the stress value for the static analysis did not exceed the permissible value related to plastic deformation. Calculations were also carried out for the value of the yield point when it was exceeded, as shown in Figure 8. The pressure load in this case was 50 bar.

## 6. Discussion

The work demonstrated a method of designing pressure vessels including a heat storage tank. Stress calculations, which were necessary during the design, were carried out with the use of normative methods and then verified with finite element analysis.

The currently available literature [34,35,36] dealt mainly with the subject of finite element analysis of tanks, without describing the methodology of their design. In addition, the paper presented a comprehensive analysis of stresses in tanks, which was missing in the above articles (e.g., the authors of [34] took into account only longitudinal stresses).

Verification by finite element analysis showed that, in the case of a tank calculated using the classical method, it was possible to maintain a safety margin of more than double for the parameters described above. The verification of the classic method with the use of a numerical code confirmed that the stresses in the tank did not pose a threat so long as the nominal pressure was not exceeded excessively. Exceeding the nominal value of the pressure in the tank by double approached, but did not theoretically exceed, the yield point. In practice, this limit could be exceeded, and the tank should not be operated at pressures higher than nominal.

The work also compared the stress requirements of three materials: carbon steel St0 (S185), stainless steel (304) and boiler steel P 265 GH. Carbon steel St0 (S185) and stainless steel (304) sufficed to make the above structure for operating pressures up to 10 bar. For each steel, the wall thickness was calculated according to the previously mentioned relationships. It was also possible to use steel dedicated to the construction of heating systems, such as P 265 GH steel, known as boiler steel, which has a higher resistance to mechanical stress. It is a steel with a low carbon content, thanks to which it exhibits high resistance to continuous operation under high pressure and temperature conditions. Under the operating conditions of heating systems, it does not crack, relax, or heat fatigue. It is also easy to weld, which is advantageous when creating permanent connections between steel elements and constructing more complex devices resistant to elevated temperatures.

## 7. Conclusions

The work showed a method of designing tanks by normative calculations and then the verification of those results by finite element analysis. Verification using the above method was cheap and enabled confirmation of results obtained during the design. This was important, as some sources describing methods of calculating tank wall thickness did not provide information such as the coefficient k of permissible stresses depending on estimated yield point or maximum destructive stress. This could lead to serious calculation errors, which could, in turn, result in the destruction of the tank.

Finite element analysis also confirmed the resistance of the structure to complex stresses, which were extensively described in this article.

Normative calculations were made for three steel grades. The calculations showed that a heat storage tank with the given dimensions, used in single-family houses, could be made of carbon or stainless steel. It would be unnecessary to use P 265 GH steel, which would significantly increase the cost of the product.

## Figures and Tables

**Table 1:** Values of α coefficient.

β	1.4	1.5	1.6	1.7	1.8	1.9	2.0
α	1.000	1.025	1.050	1.075	1.100	1.125	1.150

**Table 2:** Properties of selected steels.

Steel	Re	x [12]	k
P 265 GH	250 MPa [13]	1.65	139.9 MPa
St0	195 MPa [14]	1.8	100 MPa
St AISI 304	255 MPa [15]	1.65	142.7 MPa

**Table 3:** Calculated wall thickness for selected steels.

Steel	Wall Thickness [mm]
P 265 GH	3
St0	4
St AISI 304	3

**Table 4:** Calculated welds thickness for selected steels.

Steel	Butt Weld Thickness [mm]	Filled Weld Thickness [mm]
P 265 GH	3	3
St0	4	3
St AISI 304	3	3

**Table 5:** Parameters of the steel 304.

Parameter	Value
Density	$8027\;\frac{\mathrm{kg}}{m^{3}}$
Heat conductivity	$0.017\;\frac{\mathrm{kW}}{m\;{}^{\circ}C}$
Specific heat	$502\;\frac{J}{\mathrm{kg}\;{}^{\circ}C}$
Tensile modulus	193,053.196 MPa
Poisson ratio	0.290
Yield point	255.106 MPa
Destructive stress	579.160 MPa

## Data Availability

Not applicable.
